# Editorial: Image-Guided Radiotherapy for Effective Radiotherapy Delivery

**DOI:** 10.3389/fonc.2015.00253

**Published:** 2015-11-18

**Authors:** Nam P. Nguyen, Ulf Lennart Karlsson

**Affiliations:** ^1^Department of Radiation Oncology, Howard University, Washington, DC, USA; ^2^Department of Radiation Oncology, Marshfield Clinic, Marshfield, WI, USA

**Keywords:** cancer, computerized axial tomography, image-guided radiotherapy, comorbidity, disease-specific survival

During most of the last century, verification of patient position on the radiotherapy treatment table was considered adequate if exposed on a photographic film by a megavoltage beam. It was a general standard to expose such a film once a week, to be approved by a radiation oncologist. The latter approved it after comparison to a kilovoltage simulation film exposed at the time of initial setup of the patient before the treatment regimen started.

A common rule was to allow a ± <5 mm variation from the simulation to the treatment portal film. This often resulted in either an approval for the next week’s treatment fractions or a rejection and retake of that or the next day’s portal film. There was no film record of the next four fractions. The problems included megavoltage film resolution judged from kilovoltage simulation films as well as unrecorded possible errors for the next four fractions. Another error source was soft tissue contrast in both of these films.

The evolution of computerized axial tomography (CAT) scan from the mid-twentieth century has allowed for 3D reconstruction of the patient’s soft tissue structures by improved resolution in millimeter scan slices.

Development of the digital image visualization on computer screens now allows for fusing the reconstructed simulation image (DRR) from the CAT scanner with the mega- or kilovoltage rendering of the patient’s treatment beams. This has allowed the skilled radiotherapist to adjust the beam within a preset millimeter 3D frame to the patient’s anatomy. With this precision, a daily treatment fraction is given. The radiation oncologist can then check that body position errors have been corrected before each treatment.

Further improvement include the cone beam image obtained from the treatment accelerator and fused over the DRR, introduction of gold markers in the target volume and triangulating their positions into the simulation scan, as well as utilizing kilovoltage and or megavoltage images to attain precise beam geometry for each daily radiotherapy fraction. Another method is to use a diagnostic CAT scanner that is mechanically attached to the accelerator.

These imaging techniques are used to assure that the planned dose only covers the intended target and encompasses the IGRT concept in radiotherapy. If used properly, the precision of treatment is improved from centimeter to millimeter realms (1) and is expected to be used globally in cancer radiotherapy. Our experience is that few treatment portals need to be rejected as long as there is a requirement of immediate report to the oncologist that a specified position error has been discovered and corrected.

We consider it a necessary ingredient for clinical studies in order to measure and compare IGRT outcome data. It has the potential of not only providing better toxicity results but also to give better outcome data for patient groups who are thought to be at higher risk for toxicity, e.g., frail elderly and patients with abnormal radiosensitivity. It may also offer an avenue for dose escalation because of better organ sparing.

Our preliminary evidence is encouraging for the use of IGRT.

Elderly (>70 years of age) and younger head and neck cancer groups both tolerated definitive chemo-IGRT, without difference in grade 3–4 toxicity, treatment breaks, and with less weight loss in the elderly group (2). Another study resulted in disease-specific survival of 75% at 4 years and acceptable toxicity (3).

Elderly patients with multiple comorbidities and locally advanced rectal cancer tolerated preoperative chemo-IGRT when compared to younger patients (4). These preliminary studies suggest that IGRT may become the treatment of choice for elderly cancer patients.

Another subset of patients who may benefit from IGRT is patients with human immunodeficiency virus (HIV) infection and anal cancer. They may have an increased sensitivity to radiation because of thiol deficiency (5). Grade 3–4 skin, hematologic and gastrointestinal toxicity were frequent among HIV positive patients undergoing standard chemoradiotherapy and may result in death (6, 7). Chemo-IGRT may therefore provide HIV patients the opportunity to be treated with less toxicity (8, 9).

Finally, IGRT may allow for radiation dose escalation in cancers with high-risk for loco-regional recurrences. A recent randomized study reported a 2-year survival of 57 and 44% and local failure of 30 and 38% for locally advanced NSCLC treated to 60 and 74 Gy, respectively. The poor survival in the 74 Gy group may be associated with cardiac toxicity (10).

A 3-year survival of 45% and local failure of 15% was reported for patients with locally advanced NSCLC treated to 70–75 Gy with chemo-IGRT, with minimal toxicity (11). Dose escalation was also feasible in patients with locally advanced esophageal cancer because of lung and cardiac sparing (12).

These preliminary results are intriguing but need to be corroborated in future prospective studies.

## Author Contributions

UK and NN wrote and approved the manuscript.

## Conflict of Interest Statement

The authors declare that the research was conducted in the absence of any commercial or financial relationships that could be construed as a potential conflict of interest.
